# Immune Cell Infiltration Landscape of Ovarian Cancer to Identify Prognosis and Immunotherapy-Related Genes to Aid Immunotherapy

**DOI:** 10.3389/fcell.2021.749157

**Published:** 2021-11-03

**Authors:** Xiushen Li, Weizheng Liang, Huanyi Zhao, Zheng Jin, Guoqi Shi, Wanhua Xie, Hao Wang, Xueqing Wu

**Affiliations:** ^1^ Department of Obstetrics and Gynecology, Shenzhen University General Hospital, Shenzhen, China; ^2^ Guangdong Key Laboratory for Biomedical Measurements and Ultrasound Imaging, School of Biomedical Engineering, Shenzhen University Health Science Center, Shenzhen, China; ^3^ Shenzhen Key Laboratory, Shenzhen University General Hospital, Shenzhen, China; ^4^ Harbin Institute of Technology, Harbin, China; ^5^ Guangzhou University of Chinese Medicine, Guangzhou, China; ^6^ ZhuJiang Hospital of Southern Medical University, Guangzhou, China; ^7^ The Precise Medicine Center, Department of Basic Medical College, Shenyang Medical College, Shenyang, China; ^8^ Clinical Medical Academy, Shenzhen University, Shenzhen, China

**Keywords:** ovarian cancer, immune cell infiltration, tumor mutation burden, immunotherapy, prognosis

## Abstract

Ovarian cancer (OC) is the second leading cause of death in gynecological cancer. Multiple study have shown that the efficacy of tumor immunotherapy is related to tumor immune cell infiltration (ICI). However, so far, the Immune infiltration landscape of tumor microenvironment (TME) in OC has not been elucidated. In this study, We organized the transcriptome data of OC in the Cancer Genome Atlas (TCGA) and Gene Expression Omnibus (GEO) databases, evaluated the patient’s TME information, and constructed the ICI scores to predict the clinical benefits of patients undergoing immunotherapy. Immune-related genes were further used to construct the prognostic model. After clustering analysis of ICI genes, we found that patients in ICI gene cluster C had the best prognosis, and their tumor microenvironment had the highest proportion of macrophage M1 and T cell follicular helper cells. This result was consistent with that of multivariate cox (multi-cox) analysis. The prognostic model constructed by immune-related genes had good predictive performance. By estimating Tumor mutation burden (TMB), we also found that there were multiple genes with statistically different mutation frequencies in the high and low ICI score groups. The model based on the ICI score may help to screen out patients who would benefit from immunotherapy. The immune-related genes screened may be used as biomarkers and therapeutic targets.

## Introduction

Ovarian cancer (OC) is one of the deadliest gynecological malignancies. Owing to the lack of specific symptoms and early detection methods, approximately three-quarters of patients are already in stage III/IV at the time of diagnosis (Cheng et al., 2021). OC is the second most common cause of death in gynecological cancer. Globally, the number of deaths due to OC each year is nearly 152,000, accounting for 4.3% of all cancer deaths (Lheureux et al., 2019). In the past 30 years, the 5-year relative survival rate of most cancers has increased by one-fifth, but that of OC has not changed significantly (Siegel et al., 2018).

Cancer immunotherapy, based on the mechanism of immune escape, was rated as Breakthrough of the Year by Science (Xiang et al., 2019). Immunotherapy, including adoptive cell and checkpoint inhibitor therapy, which is often applied in the treatment lymphoma, melanoma, lung cancer, breast cancer and others, has become an indispensable method for the treatment of many cancer types (Lauss et al., 2017; Hirayama et al., 2019; Chalabi et al., 2020; Melosky et al., 2020). Tumor mutation burden (TMB), also known as tumor mutational load, is an emerging feature of cancer, which represents the number of somatic mutations (per one million bases) (Zehir et al., 2017). A growing body of literature has suggested that TMB can be used as a biomarker to identify patients suitable for immunotherapy (Steuer and Ramalingam, 2018; Klebanov et al., 2019). The tumor microenvironment (TME), composed of a variety of immune and non-immune cell populations, plays an crucial role during tumor initiation and progression. Many factors secreted within TME drive tumor biological processes such as immune suppression, pro-angiogenesis, and chronic inflammation (Pitt et al., 2016; Bader et al., 2020). The changes in the proportion of different immune cell populations and stromal cell populations in TME are related to the occurrence, metastasis, chemoresistance and progression of tumors (Turley et al., 2015). However, the overall landscape of immune cells and non-immune cells in the TME of OC is not yet clear.

In this study, we analyzed OC transcriptome data from The Cancer Genome Atlas (TCGA) and Gene Expression Omnibus databases to obtain the comprehensive outlook on 22 types of immune-related cells in the TME of OC patients through “CIBERSORT” and “estimate” packages. According to the immune cell infiltration (ICI) landscape, we divided patients with OC into 8 independent subtypes, and further established the ICI scores and immune-related gene prognosis model to predict the prognosis.

## Materials and Methods

### OV Datasets and Samples

Through the TCGA database (https://portal.gdc.cancer.gov/) and GEO array express database (https://www.ncbi.nlm.nih.gov/geo/), we obtained six data sets (GSE18520, GSE26193, GSE19829, GSE30161, GSE63885, and TCGA-OV), and collected a total of 723 OC patient samples’ transcriptome data. In order to perform unified analysis of data sets from different databases, we converted the expression value of the TCGA-OV data set into TPM (Transcripts Per Kilobase of exon model per Million mapped reads) value (Wagner et al., 2012). Furthermore, we reduced the possibility of batch effects between data sets due to non-biotechnology biases through the “ComBat” algorithm (Johnson et al., 2007).

### Consensus Clustering

We used the leukocyte signature matrix gene signature and the “CIBERSORT” algorithm to quantify the proportion of 22 immune cells in OC samples. Through the “ESTIMATE” R package, the immune and matrix cell content of each OC sample was evaluated (Yoshihara et al., 2013). We performed the “ConsensuClusterPlus” R software package based on the unsupervised clustering method to perform ICI cluster analysis on the OC data set to determine the appropriate number of clusters.

### DEGs Associated With the ICI Subgroup

With absolute multiple change> 1.5 and adjusted *p* < 0.05 as the screening conditions, the Differentially expressed genes (DEGs) between ICI subgroups were identified through the “limma” R package (Ritchie et al., 2015).

### Dimensionality Reduction and Generation of ICI Score

First, according to the expression value of DEGs of the sample, the patients in the data set were classified by the unsupervised clustering method. DEGs contained in ICI feature gene sets A and B that were positively correlated and negatively correlated with patient classification, respectively. Next, the dimensionality reduction analysis of ICI feature gene sets A and B was performed by the “Boruta” R package (Kursa and Rudnicki, 2010). Then, we obtain feature scores through principal component analysis. Finally, the ICI score of each patient was obtained through the algorithm similar to the gene expression grade index. The specific calculation formula is as follows:

$$\text{ICI score }=\sum \;{\text{PC}1}_{\text{A}}-\sum \;{\text{PC}1}_{\text{B}}\;$$

This formula was based on the Gene expression grade index algorithm, which was used to summarize the similarity between expression profile and tumor grade (Sotiriou et al., 2006).

### Functional and Pathway Enrichment Analysis

The genes in different ICI feature gene sets were respectively subjected to Gene Ontology (GO) enrichment analysis. Then, we observed the difference of the signal pathways enriched in different ICI score groups through gene set enrichment analysis (GSEA).

### Collection of Somatic Alteration Data

We downloaded and calculated the number of non-synonymous mutations in TCGA-OV patients. According to the ICI scores, the somatic mutations of the driver genes in different groups of patients were evaluated accordingly. We identified the driver genes in OC patients by “maftool” package. The top 20 driver genes with the highest mutation frequency were analyzed, respectively.

### Analyze the Predictive Performance of the ICI Scores and TMB Model

We constructed the Receiver Operating Characteristic (ROC) curve to analyze the predictive performance of the ICI score model. The TMB and ICI score models were combined through univariate cox (uni-cox), lasso, and multivariate cox (multi-cox) analysis, and ROC curve and Kaplan-Meier curve were used to analyze its prediction performance.

### Construction and External Verification of Prognostic Models of Immune-Related Genes

Uni-cox analysis was used to screen out the ICI characteristic genes related to the patient’s prognosis. The lasso regression algorithm was used to delete the genes with higher correlation to prevent overfitting. We constructed the prognostic model through multi-cox analysis. The OC data (GSE140082) was used for external verification. The ICI scores, TMB and immune genes were used to construct a prognostic model (uni-cox, lasso, multi-cox).

### Statistical Analyses

All statistical analyses were performed by SPSS Version 21.0 software. The data comparison between the two groups used unpaired Student’s t test for statistical significance, and the non-normally distributed variables used Mann-Whitney *U* test. For the data comparison between the above two groups, the parametric method and the non-parametric method used one-way analysis of variance and Kruskal-Wallis test, respectively. The correlation coefficient was calculated by Spearman correlation analysis and distance correlation analysis. The survival curve of each subgroup was drawn by Kaplan-Meier method. The log-rank test was used to evaluate whether the difference is statistically significant. Only when the two-tailed *p*-value is < 0.05, the difference is considered to be statistically significant.

## Results

### Landscape of OC TME

We converted the expression value of the TCGA-OV data set to TPM value and merged the data of GSE18520, GSE26193, GSE19829, GSE30161, and GSE63885 (Supplementary Table S1). The ratio of 22 immune cells in OC samples was calculated by executing the CIBERSORT algorithms (Supplementary Table S2). The “ConsesusClusterPlus” R package clustered OC patients into 8 subtypes through an unsupervised clustering method (Supplementary Figure S1).

**FIGURE 1:**
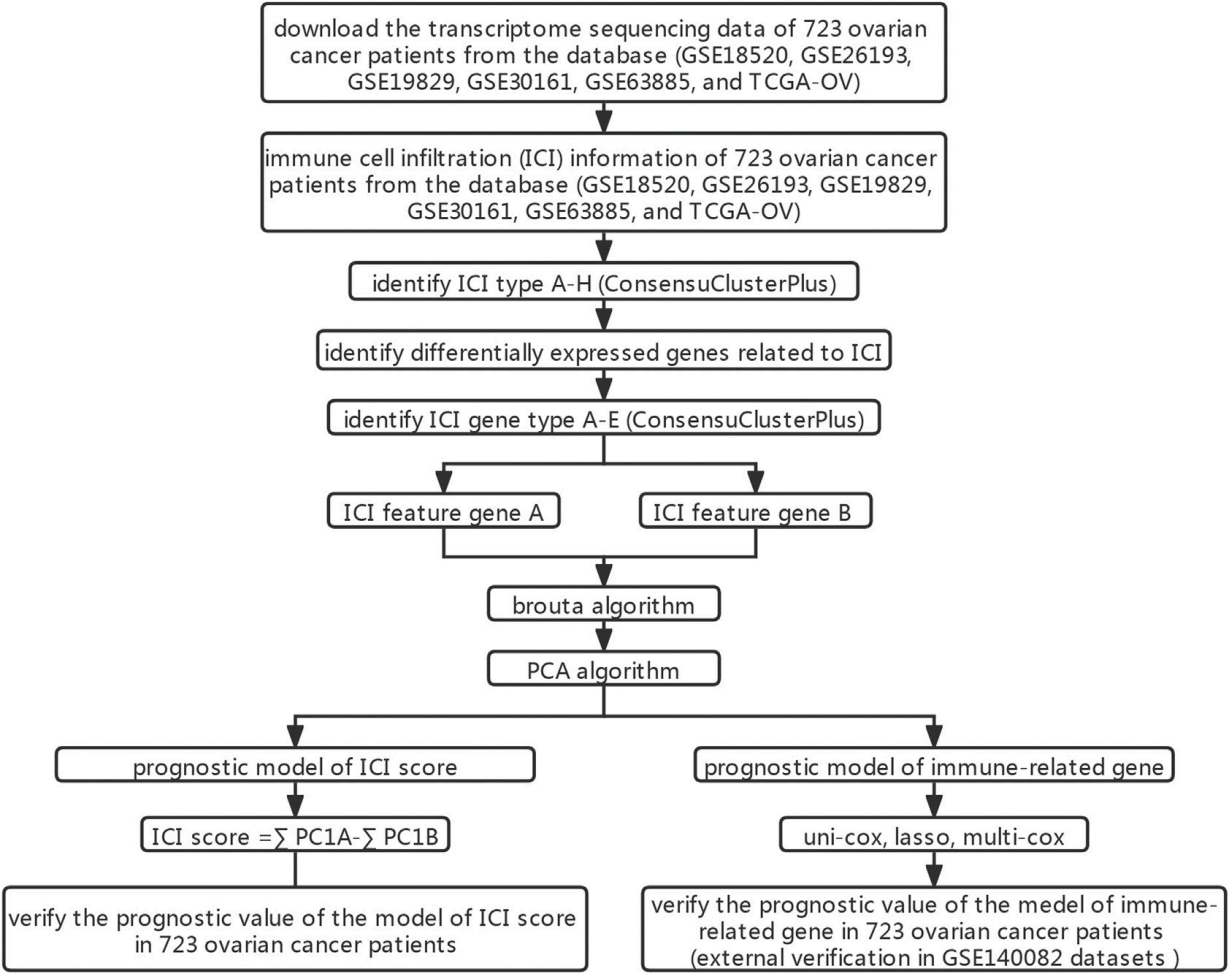
Flow chart.

**FIGURE 2:**
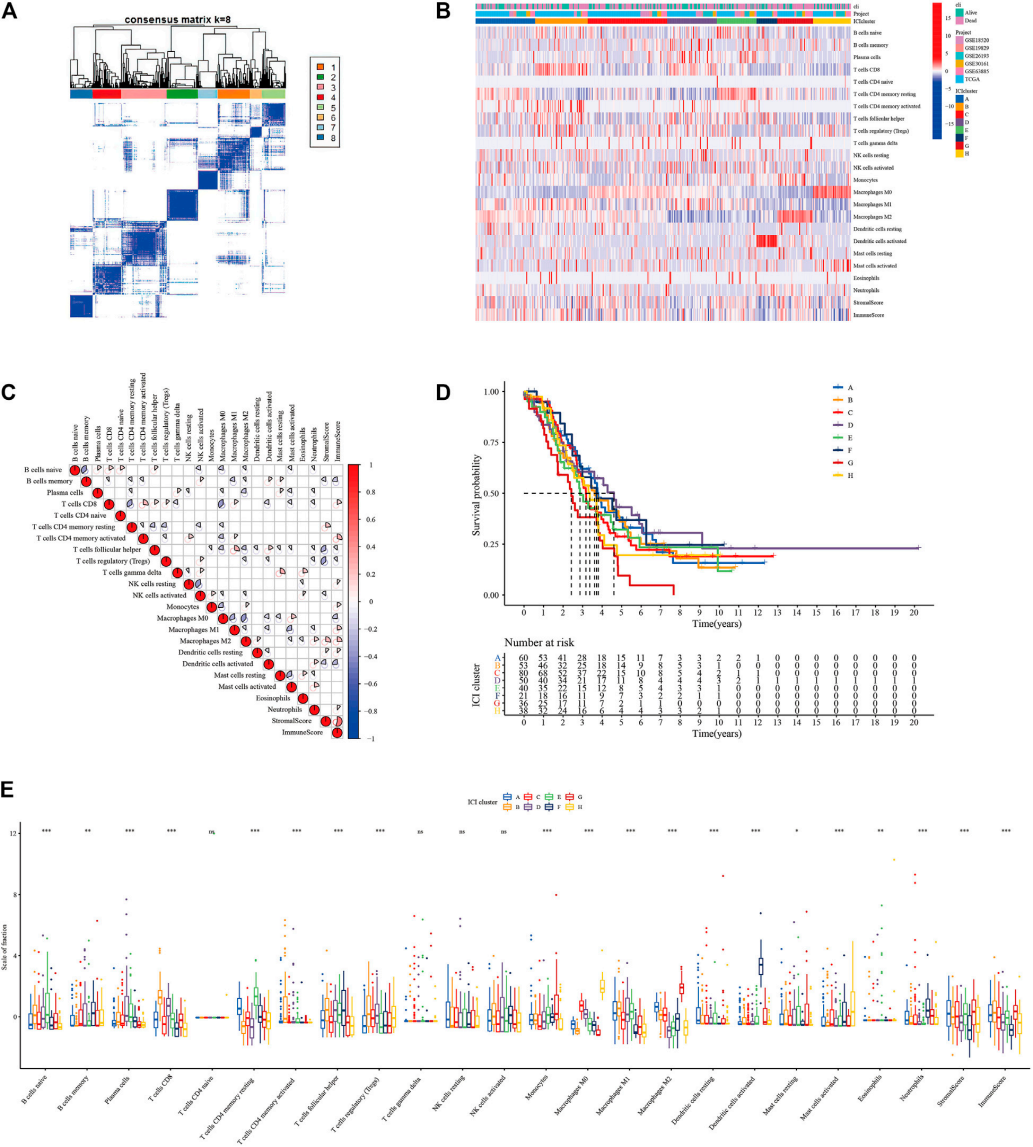
Infiltration of immune cells in TME of patients with OC (GSE18520, GSE26193, GSE19829, GSE30161, GSE63885, and TCGA-OV). **(A)** Consensus matrixes of all OC samples (k = 8). **(B)** Unsupervised clustering heat maps of tumor-infiltrating immune cells. **(C)** Correlation analysis between 22 tumor-infiltrating immune cell types. **(D)** Kaplan-Meier overall survival curve of the 8 ICI subtypes. **(E)** Difference analysis of 22 tumor-infiltrating immune cell types in 8 ICI subtypes.

Figure 1 In order to clarify the inherent biological differences of different clinical phenotypes of OC samples, We divided patients into 8 ICI subgroups (Figure 2A). Figure 2B showed the distribution of 22 types of tumor cells in 8 ICI subgroups through the heat map. The correlation between the 22 types of tumor cells was further analyzed, and a map of correlation coefficient was constructed (Figure 2C). As shown in Figure 2D, the median survival time of each subtype was different. ICI subtype D patients had the longest median survival period, which was characterized by high infiltration of T cells regulatory, NK cells activated, plasma cells, and T cells follicular helper. The median survival time of patients with ICI subtype G was the shortest, which was characterized by the highly infiltrating cells Macrophages M2, Neutrophils, and Monocytes. Further analysis of the differences in the proportion of 22 types of tumor cells among the 8 ICI subtypes revealed that only in the four types of cells were no statistical difference between the subgroups (Figure 2E).

### Identify Immune Gene Subtypes and GO Enrichment Analysis

**FIGURE 3:**
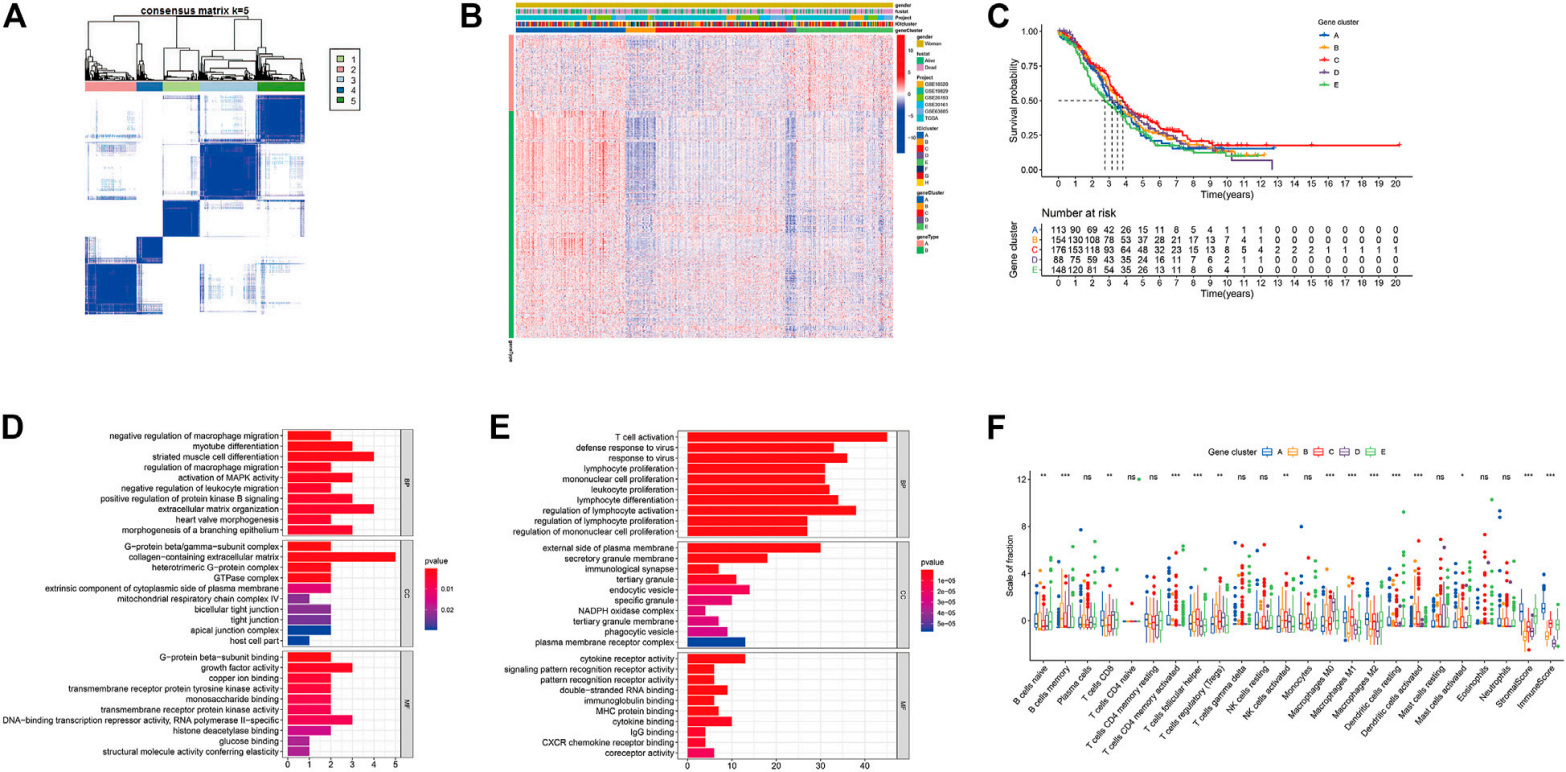
Identification of immunogenic gene subtypes. **(A)** Consensus matrixes of all OC samples (k = 5). **(B)** Unsupervised clustering of common DEG among 5 ICI cluster groups. **(C)** Kaplan-Meier curve of the 5 groups of patients. **(D)** GO enrichment analysis of ICI feature gene set A. **(E)** GO enrichment analysis of ICI feature gene set B. **(F)** The proportion of tumor infiltrating immune cells in the 5 gene clusters (*p < 0.05; **p < 0.001; ***p < 0.001; ns means statistical difference).

We screened the differentially expressed genes among eight immune subtypes by “limma” R software. According to the obtained 817 differentially expressed genes, the OC patients were divided into five gene subgroups by using the “ConsensuClusterPlus” R package based on the unsupervised clustering algorithm (Supplementary Figure S2, Figure 3A). As shown in Figure 3C, we found patients in the ICI gene C group had the best prognosis, while those in the E group had the worst.

The 205 genes positively related to the gene subgroup were called ICI gene signature set A, while the 612 genes positively related to the gene subgroup were called ICI gene signature set B (Supplementary Table S3). Drew the expression of these 817 characteristic genes in OC samples through the “pheatmap” R package (Figure 3B). We performed dimensionality reduction analysis to reduce redundant genes, and finally got 37 and 237 genes, respectively (Supplementary Table S4). Through “clusterProfiler” R package we performed GO and Kyoto Encyclopedia of Genes and Genomes (KEGG) enrichment analysis on the ICI gene signature sets A and B after dimensionality reduction analysis (Figures 3D,E, Supplementary Figures S3E–F). Supplementary Table S5 provides detailed GO and KEGG enrichment analysis results. As shown in Figure 3F, the ICI gene subgroup C with the best prognosis showed the highest proportion of T cell follicular helper, monocytes, and macrophages M1, while the ICI gene subgroup E with the worst prognosis showed the highest proportion of naive B cells and the lowest proportion of regulatory T cells.

### Construction of the ICI Score

**FIGURE 4:**
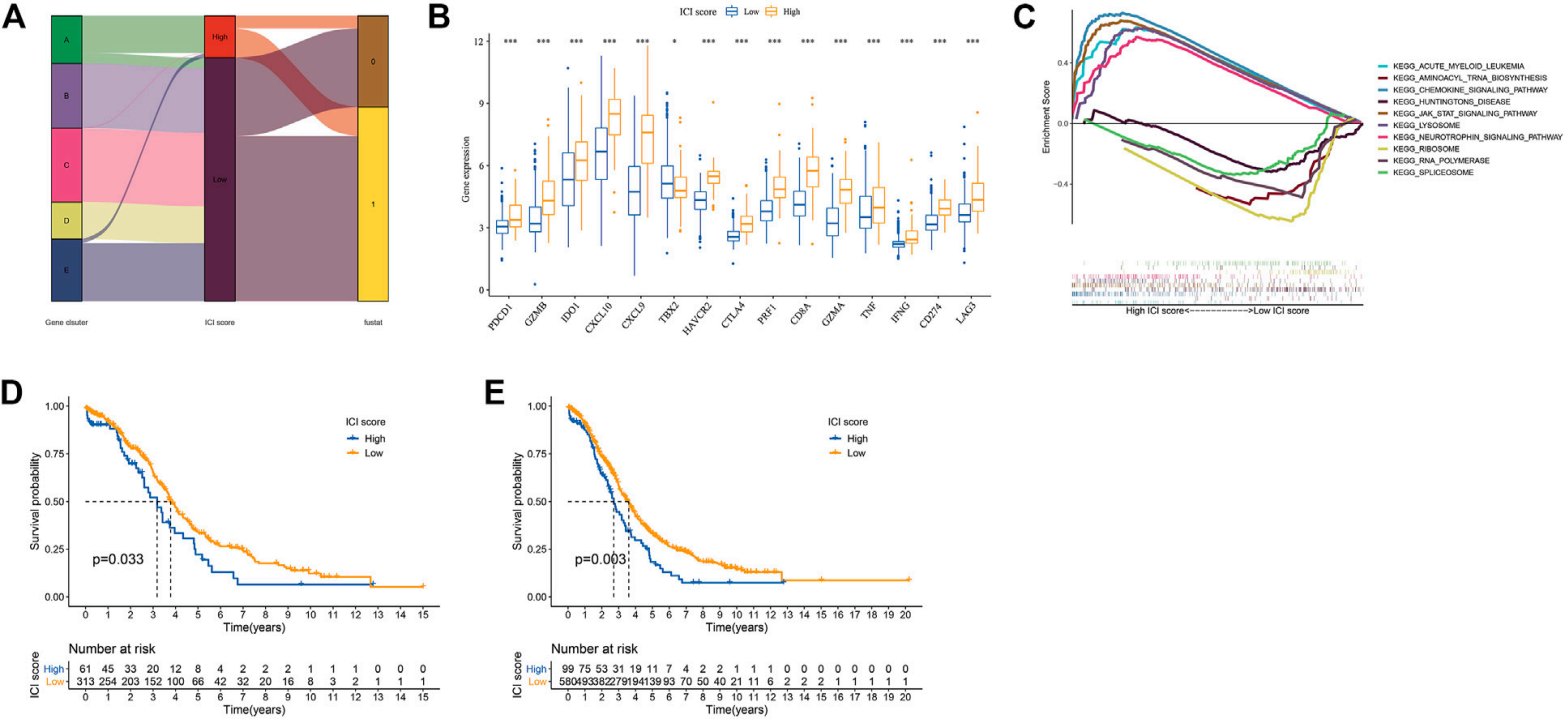
Construction of ICI scores model. **(A)** Alluvial plots of the distribution of ICI gene clusters in different ICI clusters, ICI scores and survival outcome groups. **(B)** The expression of the Immune related genes (*p < 0.05; ***p < 0.001). **(C)** GSEA enrichment analysis (only the top five are shown). **(D)** Kaplan-Meier curve (TCGA-OC). **(E)** Kaplan-Meier curves (GSE18520, GSE26193, GSE19829, GSE30161, GSE63885, and TCGA-OV).

We used principal component analysis (PCA) algorithm to analyze the ICI landscape of patients with OC. After calculation, we finally got the ICI prognostic landmark score. According to the ICI scores of patients with OC, we used the “survminer” R package to find the best cut-off value, and divided the patients into two groups with high and low scores. Then, the expression of immune-related genes in the two groups with high and low ICI scores was searched. The distribution of patients in five gene clusters was represented in Figure 4A. In this study, we selected the immune checkpoint-related genes and the immune activity-related genes for display. As shown in Figure 4B, the expressions of these 15 genes were statistically different between the two groups. In addition, the results of GSEA showed that in the high ICI group, jak-stat and chemokine signaling pathways were significantly enriched, while in the low ICI group, RNA polymerase and ribosome signaling pathways were significantly enriched (Figure 4C; Supplementary Table S6). We analyzed the relationship between the patient’s ICI scores and survival time through the “survival” R package, and found that in the OC patient cohort of the TCGA database, patients with high ICI scores had poorer prognosis (Figure 4D). Further we analyzed the relationship between the ICI scores and prognosis of all OC patients included in this study and found that was consistent with the relationship obtained by TCGA-OC patients (Figure 4E).

### TME Characteristics of the TCGA Subtype and Cancer Somatic Genome

**FIGURE 5:**
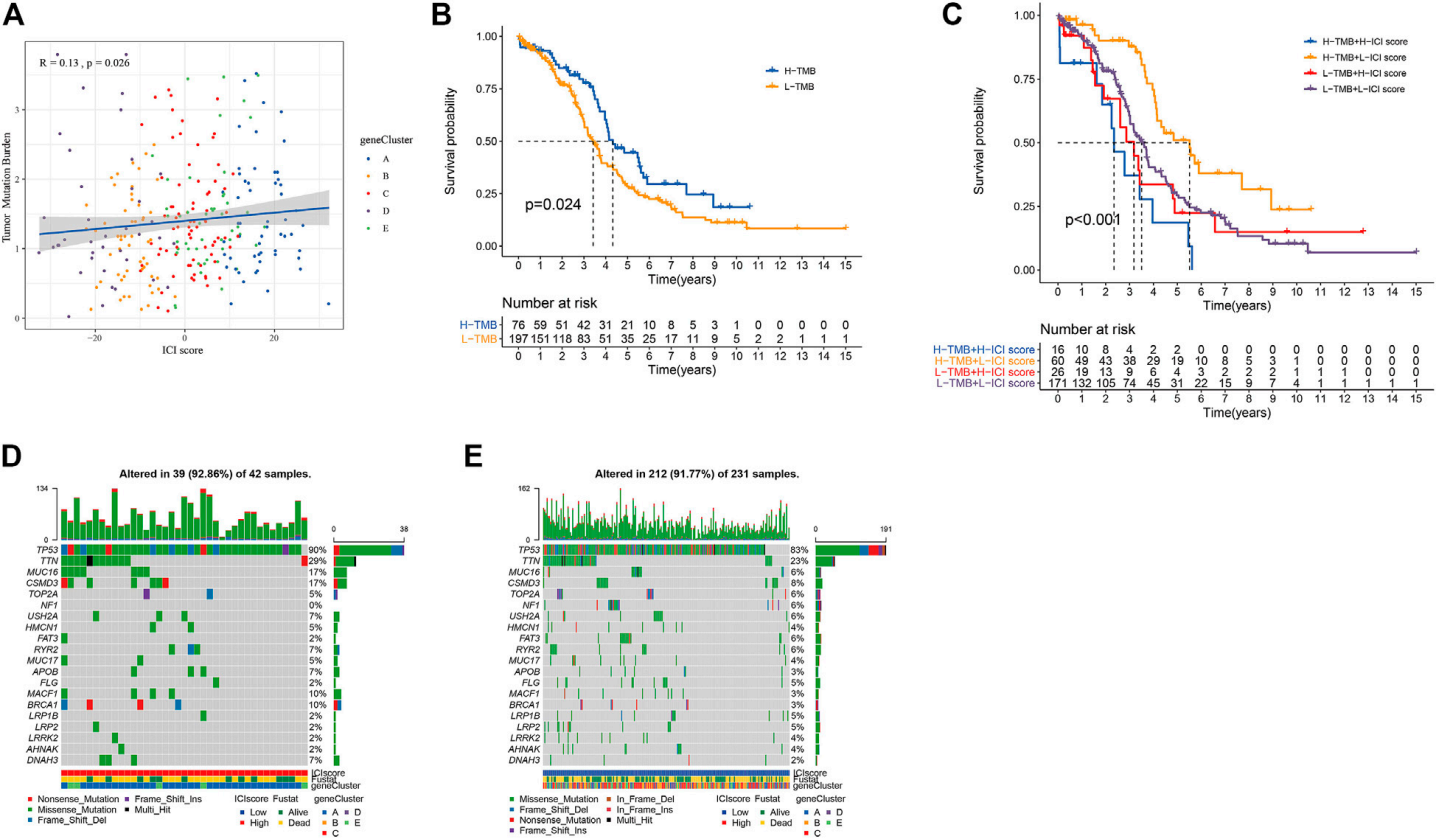
The results of ICI score and somatic variation. **(A)** The scatter plot of the ICI score and mutation load (*p* = 0.0026). **(B)** Kaplan-Meier curve of the high TMB group and the low TMB group (TCGA-OC, *p* = 0.024). **(C)** Kaplan-Meier curve of OC patients (TCGA-OC, *p* < 0.001, use TMB mutation load and ICI score to stratify patients). **(D,E)** are, respectively oncoPrint constructed based on ICI score.

At present, Immune Checkpoint Blocking (ICB) therapy had been applied to a variety of tumor diseases, improving the overall survival rate of patients (Hodi, 2010; Borghaei et al., 2015; Nghiem et al., 2016; Tom, 2018). A number of studies had shown that to mutation. Tumor mutation burden (TMB) could be used to predict the efficacy of ICB, and it has become a biomarker for various cancer types to identify patients who will benefit from immunotherapy (Rizvi et al., 2015; Timothy et al., 2015; Hugo et al., 2016; Carbone et al., 2017; Chan et al., 2019). Based on the important clinical significance of TMB for immunotherapy (Supplementary Table S7), we further explored the inner link between TMB and ICI scores to clarify the genetic information of ICI subgroups. Correlation analysis revealed a positive correlation between TMB and ICI scores (Spearman coefficient: *r* = 0.13, *p* = 0.026; Figure 5A). We found the best cutoff value by “survminer” R package, and divided the patients into high and low groups. By analyzing the relationship between TMB and patient prognosis, we found that the prognosis of patients in the high TMB group was better (*p* = 0.024; Figure 5B). We combined the patient’s TMB and ICI scores information for analysis. It was found that patients with high ICI score and low TMB group had the best prognosis, while patients with high ICI score and high TMB group had the worst (*p* < 0.001; Figure 5C).

We obtained the driver genes of OC and evaluated the somatic mutations of patients in different ICI subgroup. Figures 5D,E showed respectively the mutation distributions of the 20 driver genes with the highest frequency of changes in the high and low ICI subgroups. These results might provide new directions for studying the mechanisms of immunotherapy, gene mutations, and tumor ICI distribution.

### The Predictive Performance of the ICI Scores and TMB Model

We found that the prediction performance of the ICI scores model was poor by establishing the ROC curve (Supplementary Figures S3A,B). Further analysis of the predictive performance of the ICI scores and TMB combined model, through ROC curve and Kaplan-Meier curve found that its predictive performance was average (Supplementary Figures S3C,D).

### Construction and External Verification of Prognostic Models of Immune-Related Genes

**FIGURE 6:**
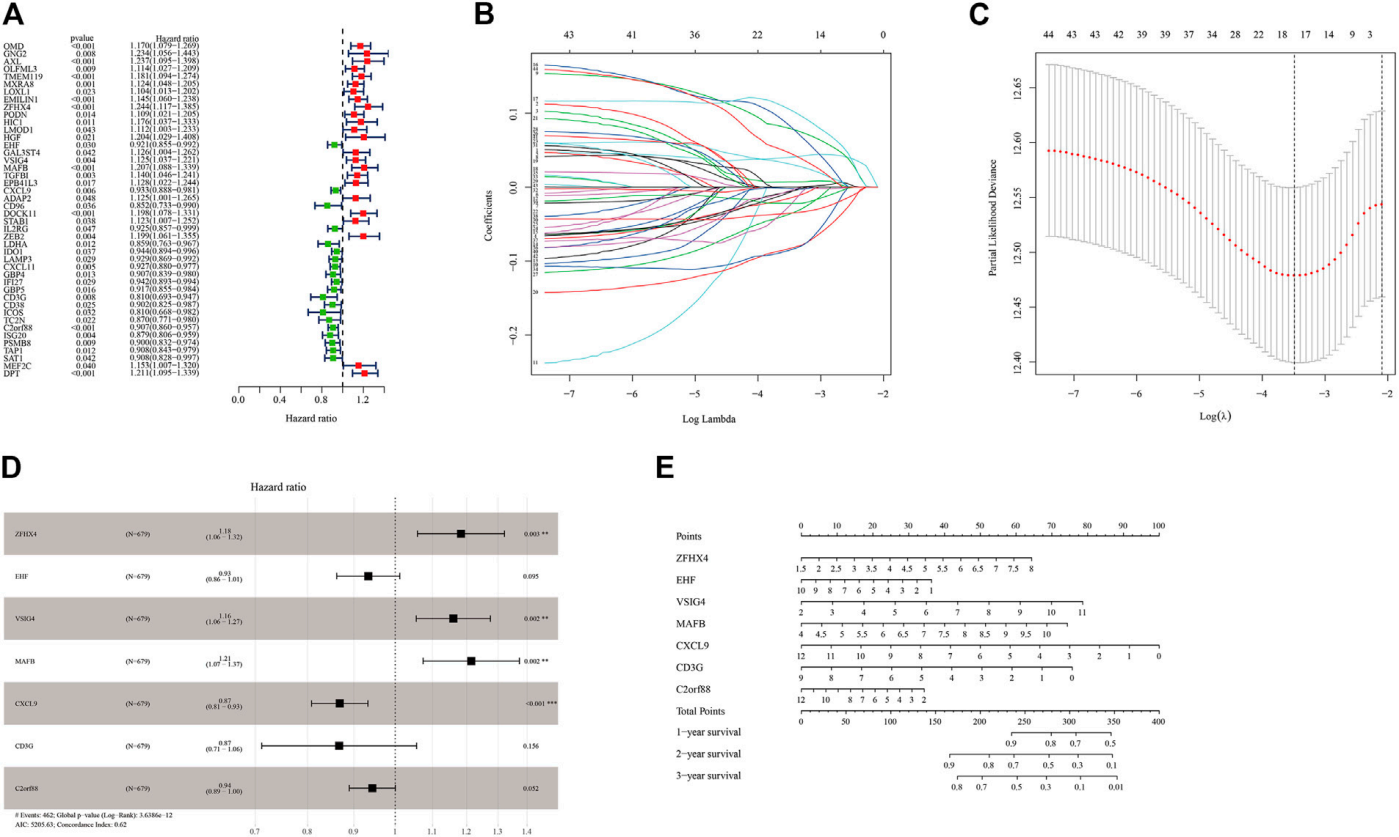
Construction of the prognostic model of immune-related genes. **(A)** Uni-cox variance analysis of ICI feature gene set. **(B,C)** Lasso regression analysis results for genes obtained from uni-cox analysis. **(D)** Multi-cox analysis results of genes obtained from lasso regression analysis. **(E)** The nomogram of the prognostic model.

Through uni-cox analysis, a total of 44 genes related to the prognosis of OC patients and immunity were obtained (Figure 6A). In order to prevent overfitting, the lasso analysis was used to further screen the genes related to the patient’s prognosis. As shown in Figures 6B,C, the value of the intersection point corresponding to the log(λ) of −3.5 was the smallest, and the corresponding values were 18. Therefore, we screened out 18 genes for subsequent analysis. The multi-cox analysis was performed on these 18 genes, and the results were shown in Figure 6D. The results of the multi-cox analysis were displayed in the nomogram (Figure 6E). The results of each step of the analysis were saved in Supplementary Table S8.

**FIGURE 7:**
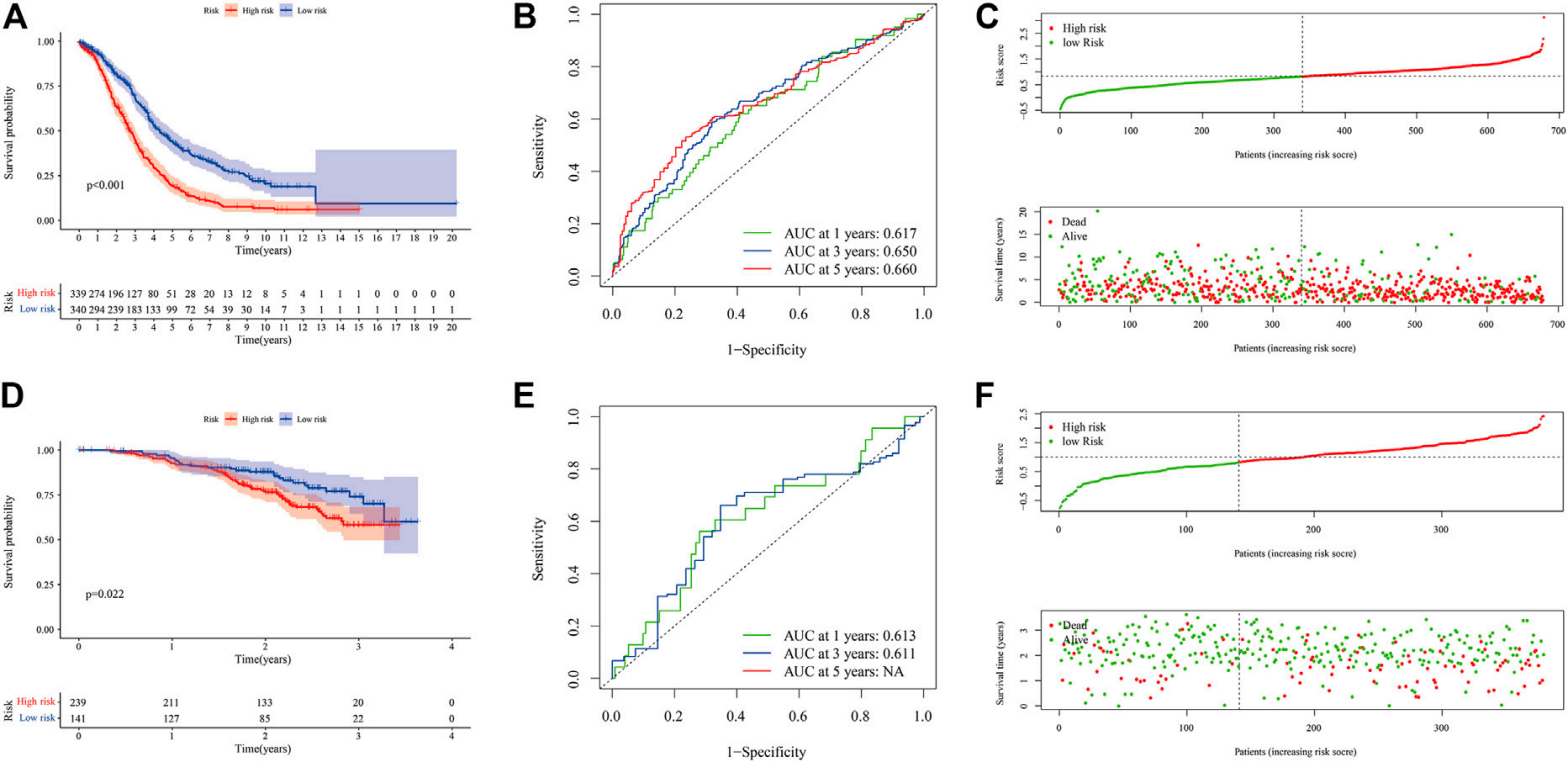
Evaluation and external verification of prognostic models of immune-related genes. **(A–C)** show the analysis result of GSE18520, GSE26193, GSE19829, GSE30161, GSE63885, and TCGA-OV data set. **(A)** Kaplan-Meier curve (6 independent OC data sets). **(B)** ROC curves. **(C)** Scatter plot of the risk score and overall survival. **(D–F)** show the analysis result of GSE140082. **(D)** Kaplan-Meier curve (external verification). **(E)** ROC curves (external verification). **(F)** Scatter plot of patient risk score and overall survival (external verification).

We tested the prognostic model of immune-related genes. The survival curve showed that the prognosis of patients in the low-risk group was better, and there were statistical differences between the groups (Figure 7A). We observed the forecasting performance of the model through the ROC curve. As the forecasting time increased, the forecasting effect of the model showed an upward trend (Figure 7B). We sorted the samples of OC patients according to the risk score and found that as the score increased, the risk of death was higher (Figure 7C). We also obtained good prediction performance in the externally verified GSE140082 data set (Figures 7D–F; Supplementary Table S9).

Combining ICI scores, TMB, and immune genes (uni-cox, lasso, multi-cox), we further built a prognostic model (Supplementary Figure S4), and found that the prediction performance of this model was basically the same as that of immune-related gene models. The results of each step of the analysis were saved in Supplementary Table S10.

## Discussion

Cancer is a global public health problem and the second leading cause of death (Siegel et al., 2020; Huang et al., 2021b). In the last few years, a variety of specific immunotherapy drugs have been used in the clinical treatment of cancer patients. Immunotherapy, which uses the patient’s immune capacity to treat cancer and prevent recurrence, has become the first-line treatment for some types of tumors (Kruger et al., 2019; Szeto and Finley, 2019). Immunotherapy has made remarkable progress in the treatment of tumors, but it still faces many challenges. The first obstacle is that only a few patients can benefit from immunotherapy. In 2018, it was estimated that two-fifths of the United States patients treated tumors with immune checkpoint inhibitor-related drugs, but the immune response rate was only 13% (Haslam and Prasad, 2019). Drug resistance might appear during chemotherapy, which greatly reduces the efficacy of treatment (Huang et al., 2021a). In this study, we established the model for predicting the immune benefit of OV patients and the immune prognosis evaluation model, which may provide help for immunotherapy of patients with ovarian cancer.

OC is classified as “immunogenic tumor”. Immune cell populations in tumors, peripheral blood and ascites fluid, including T and B lymphocytes, natural killer cells, etc., have great significance in the treatment of OC (Santoiemma et al., 2016; Yang et al., 2020). Non-immune cells in the TME may also have an impact on the efficacy of immunotherapy (Rodriguez et al., 2018). We divide the samples into eight different immune subtypes based on the content of 22 immune cells in OC patients. Further analysis found that the proportion of 18 immune cell populations between immune subtypes was statistically different. The proportion of follicular helper T cells, M1 macrophages, M2 macrophages, mast cells activated and the established ICI score model had the strong correlation with prognosis (Supplementary Figure S5). The tumor suppressor effect of follicular helper T cells was related to its ability to promote B cell maturation, affinity maturation and antibody secretion (Bindea et al., 2013; Crotty, 2014; Gu-Trantien et al., 2017). In addition, follicular helper T cells could promote the survival of CD8 T cells related to the prognosis of OC patients by secreting IL-21 (Zeng et al., 2005; Søndergaard et al., 2010; Kroeger et al., 2016). M1 macrophages could promote T cell immunity and played an anti-cancer effect, while M2 macrophages had the anti-inflammatory effect and played the cancer-promoting effect (Galli et al., 2011; Tamura et al., 2018). The relative proportion of M1 and M2 macrophages were closely related to the immunity of T cells, which had a major influence in the immune checkpoint blocking therapy of tumors (Galli et al., 2011). Mast cells in the tumor mass could inhibit tumor proliferation and angiogenesis, but their presence in the area around the tumor might promote tumor progression (Johansson et al., 2010). The positive and negative regulatory relationships between these four types of cells and tumor progression were opposite to the coefficient of multi-cox analysis, which was consistent with the conclusion that patients with low ICI scores in this study had a better prognosis.

Differences in gene expression during tumor formation might lead to changes in information transmission between immune cells, thereby affecting the occurrence of immune responses in the human body (Chen and Mellman, 2017). In this study, in order to have more in-depth understanding of the gene characteristics related to the immune system in patients with OC, we first extracted immune-related genes and performed the cluster analysis on patients with OC. ICI gene cluster C with the highest Macrophages M1 and T cells follicular helper content had the best prognosis. We speculated that OC patients with ICI gene cluster C could benefit from immunotherapy, and the gene cluster used in this study may provide new targets for precision mmunotherapy. ICI gene cluster E with higher Macrophages M2 and lower T cells follicular helper content had the worst prognosis. These were consistent with the results of the previous multi-cox analysis of the proportion of 22 immune cells. Therefore, we speculated that macrophage M1, macrophage M2, and T cell follicular helper cells might have important implications for the immunotherapy of OC.

Due to the large differences in individual immune environments, we quantified the ICI score of patients with OC. We used dimensionality reduction analysis on ICI gene feature sets A and B. The GO enrichment analysis results of ICI gene feature sets A and B were related to macrophages and T cells, respectively, which played extremely important roles in immunotherapy. Through GSEA, we discovered that chemkine, neurotrophin, and jak-stat signaling pathway might play significant roles in the occurrence and development of OC. Clinical studies have found that gene mutations were related to the clinical benefits of immunotherapy and could be used as potential biomarkers for immunotherapy (Wang et al., 2019; Pan et al., 2021). Recently, the United States Food and Drug Administration approved pembrolizumab as a clinical drug for high TMB-H solid tumors (≥10). Survival analysis found that TMB was related to patient prognosis. The joint analysis of TMB and ICI scores found that patients with high TMB and low ICI score had the best prognosis, while patients with H-TMB and high ICI score had the worst. It meant that ICI score and TMB might play a role in different aspects of OC immunotherapy. The low correlation between ICI score and TMB also confirmed this statement.

## Conclusion

By analyzing the ICI characteristics of OC patients, we found that follicular helper T cells, mast cell activation, M1 macrophages and M2 macrophages may affect the patient’s immunotherapy, and established an ICI score to predict the patient’s clinical benefit Provide a basis. We have further identified immune-related genes, which can be used as biomarkers for immunotherapy evaluation and targets for personalized immunotherapy.

## Data Availability

The datasets presented in this study can be found in online repositories. The names of the repository/repositories and accession number(s) can be found in the article/Supplementary Material.

## Abbreviations

OC, ovarian cancer; ICI, immune cell infiltration; TME, tumor microenvironment; TCGA, the cancer genome atlas; GEO, gene expression omnibus; multi-cox, multivariate cox; TMB, tumor mutation burden; TPM, transcripts per kilobase of exon model per million mapped reads; GO, gene ontology; GSEA, gene set enrichment analysis; ROC, receiver operating characteristic; uni-cox, univariate cox; KEGG, kyoto encyclopedia of genes and genomes; PCA, principal component analysis; ICB, immune checkpoint blocking.
