# Supplementary figures and images for: Immune Cell Infiltration Landscape of Ovarian Cancer to Identify Prognosis and Immunotherapy-Related Genes to Aid Immunotherapy
Source: Front Cell Dev Biol. 2021 Nov 3;9:749157. doi: 10.3389/fcell.2021.749157 (PMC8595115; doi:10.3389/fcell.2021.749157)

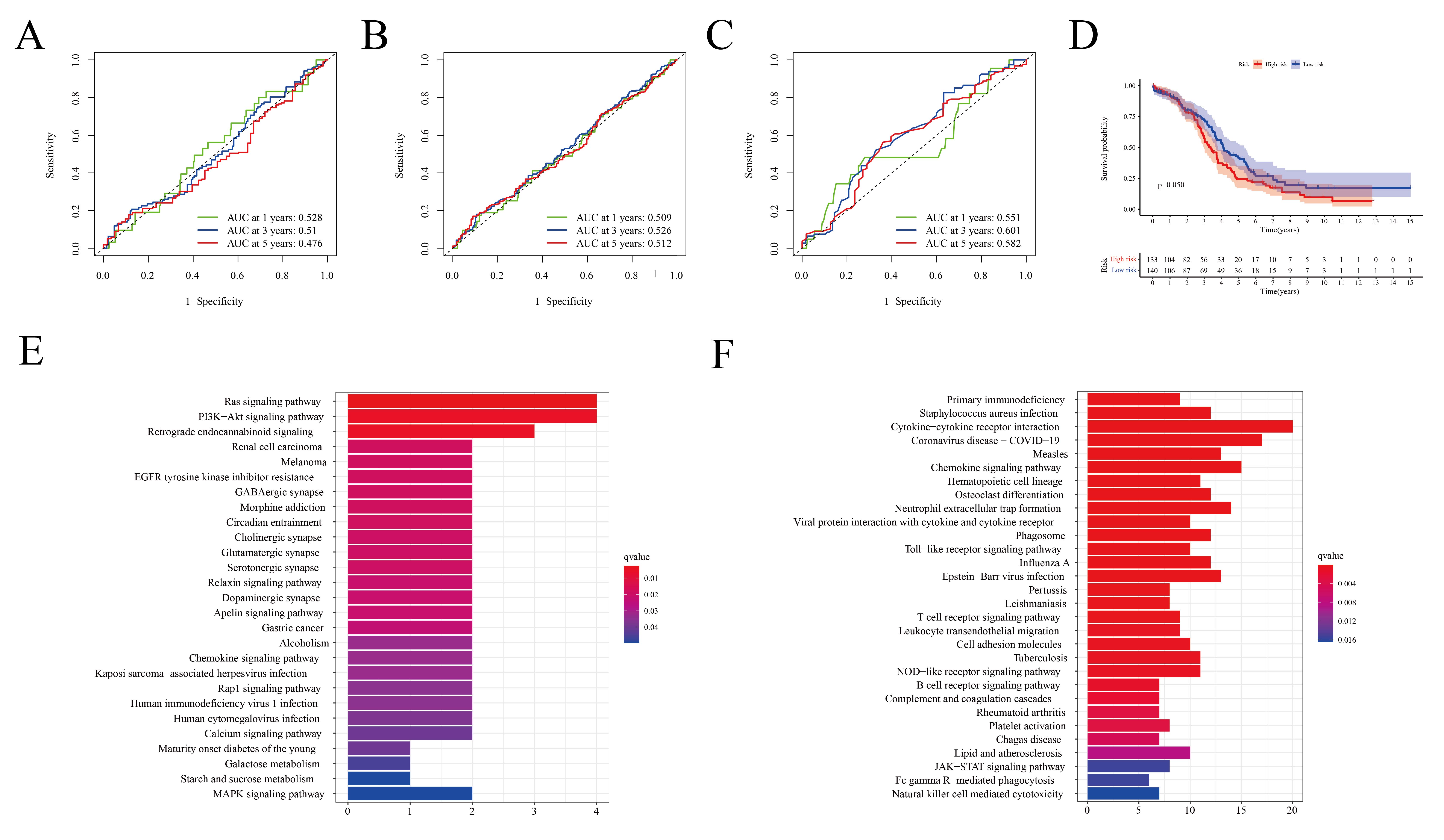

Supplement: Supplementary file 2 [file Image3.JPEG]

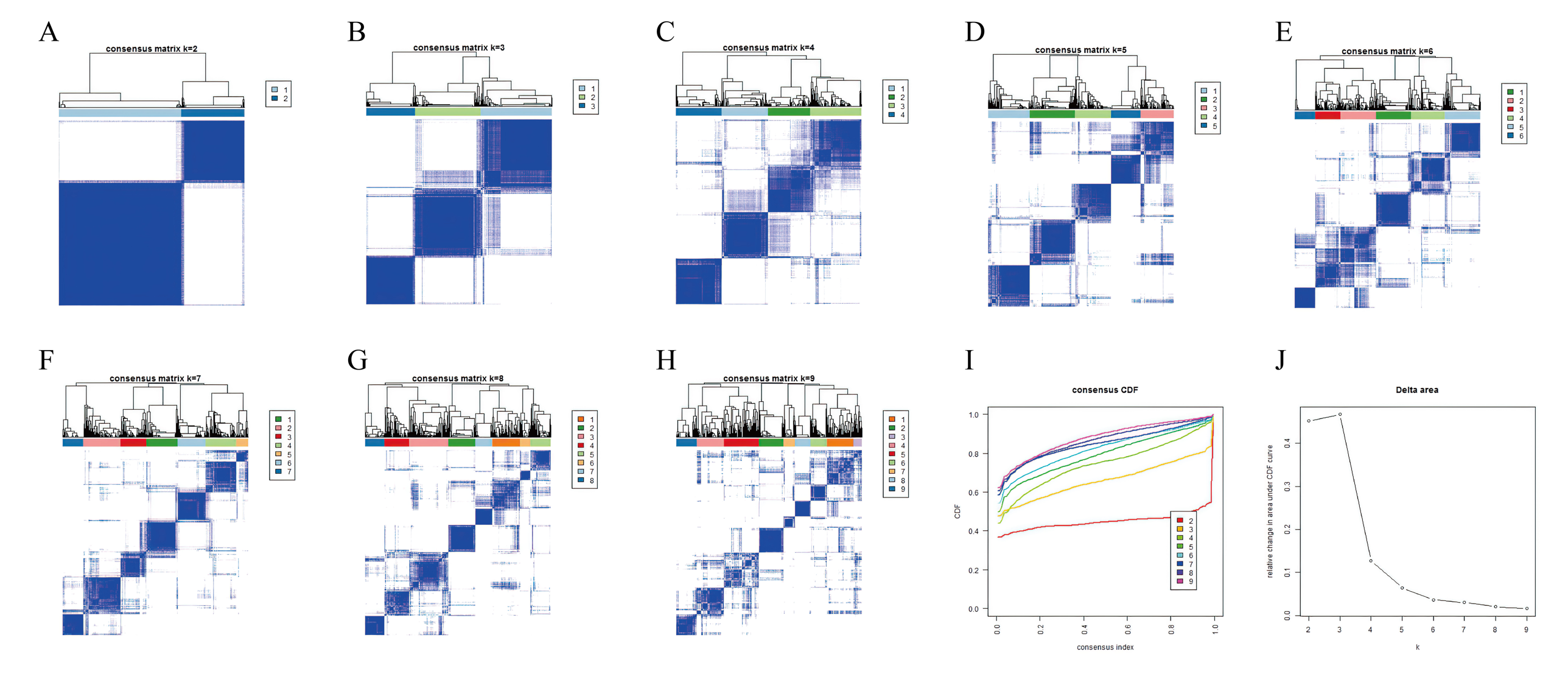

Supplement: Supplementary file 6 [file Image1.JPEG]

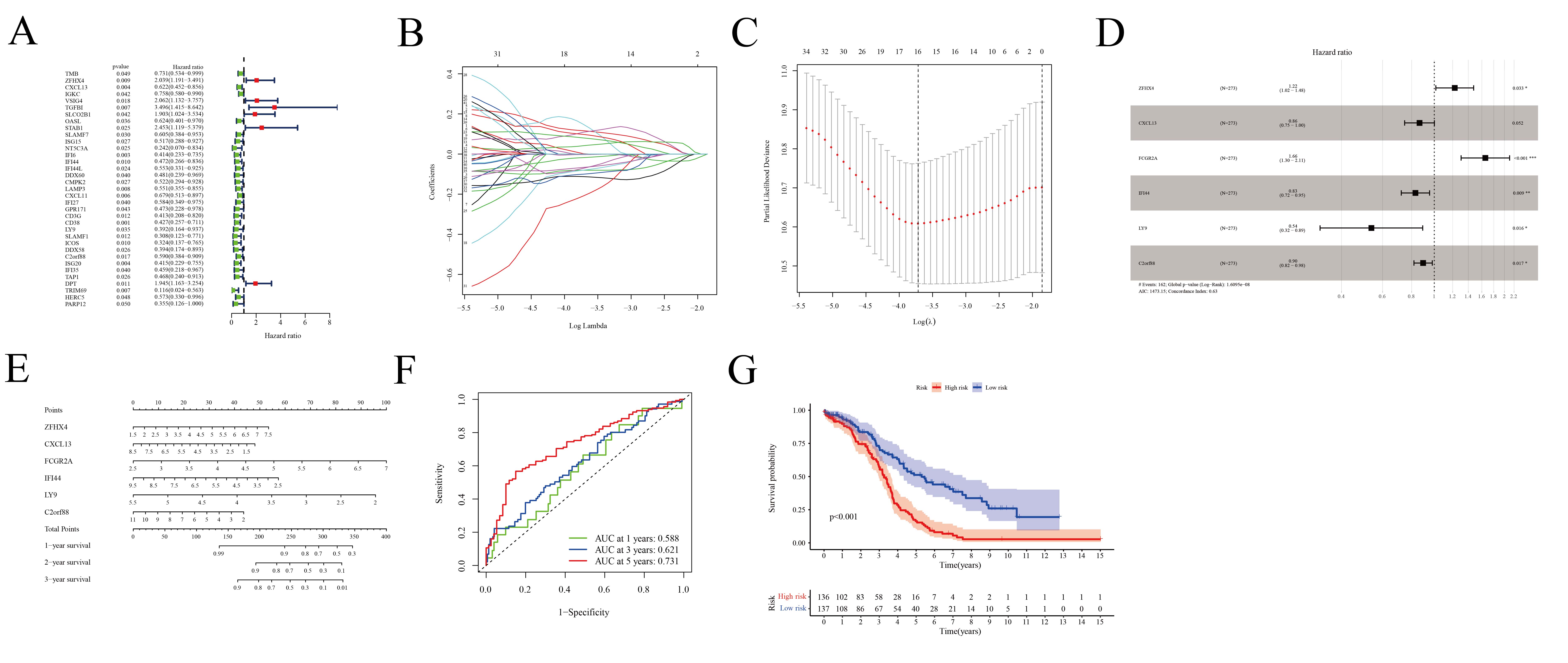

Supplement: Supplementary file 7 [file Image4.JPEG]

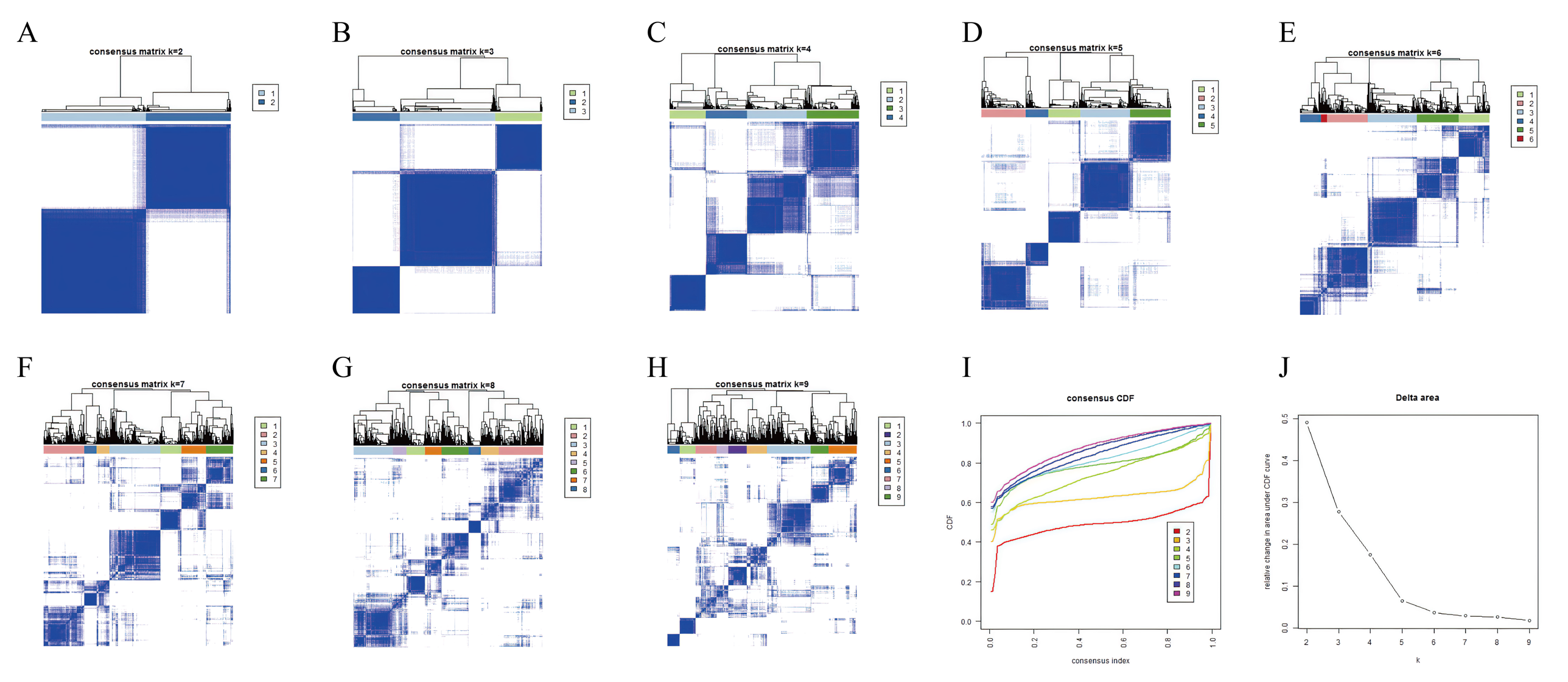

Supplement: Supplementary file 8 [file Image2.JPEG]

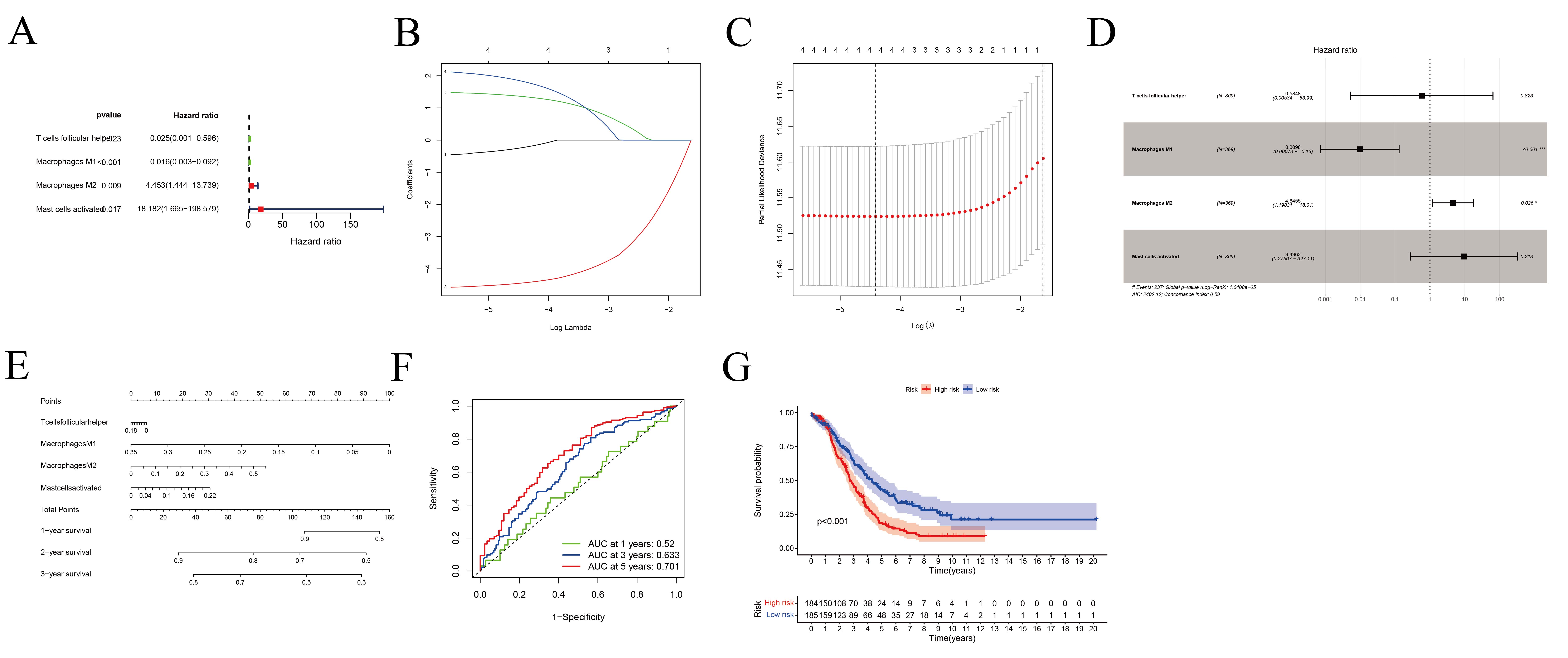

Supplement: Supplementary file 9 [file Image5.JPEG]
