# Supplementary material for: Immune Cell Infiltration Landscape of Ovarian Cancer to Identify Prognosis and Immunotherapy-Related Genes to Aid Immunotherapy
Source: Front Cell Dev Biol. 2021 Nov 3;9:749157. doi: 10.3389/fcell.2021.749157 (PMC8595115; doi:10.3389/fcell.2021.749157)
Supplement: Supplementary file 4 [file Table1.DOCX]

**Supplementary Figure Legends**

**Supplementary Figure 1.** Analysis of ICI subtypes of OC samples. (A)-(H) Consensus matrix of ICI subtypes of all OC samples for each k (k = 2-9), displaying the clustering stability by using 1000 iterations of hierarchical clustering. (I) Consensus among subtypes for each cluster number k. (J) The Delta area curve used for consensus clustering represents under the CDF curve the relative change in the area for each cluster number k compared to k-1.

**Supplementary Figure 2.** Analysis of gene subtypes of OC samples. (A)-(H) Consensus matrix of ICI subtypes of all OC samples for each k (k = 2-9), displaying the clustering stability by using 1000 iterations of hierarchical clustering. (I) Consensus among subtypes for each cluster number k. (J) The Delta area curve used for consensus clustering represents under the CDF curve the relative change in the area for each cluster number k compared to k-1.

**Supplementary Figure 3.** Analysis of prognostic models of ICI score and TMB. (A) ROC curves of patients with OC at 1, 3, and 5 years (6 independent OC data sets; prognostic models of ICI score). (B) ROC curves of patients with OC at 1, 3, and 5 years (TCGA OC data sets; prognostic models of TMB). (C) ROC curves of patients with OC at 1, 3, and 5 years (TCGA OC data sets; prognostic models of ICI score and TMB). (D) Kaplan-Meier curve of patients with OC (TCGA OC data sets; prognostic models of ICI score and TMB). (E) KEGG enrichment analysis of ICI feature gene set A. (F) KEGG enrichment analysis of ICI feature gene set B.

**Supplementary Figure 4.** Construction of the prognostic model of immune-related genes, ICI score and TMB. (A) The results of uni-cox variance analysis. (B) and (C) The results of lasso regression analysis. (D) The results of multi-cox analysis results of genes obtained from lasso regression analysis. (E) The nomogram of the prognostic model. (F) ROC curves of patients with OC at 1, 3, and 5 years (TCGA OC data sets). (G) Kaplan-Meier curve of patients with OC (TCGA OC data sets).

**Supplementary Figure 5.** Construction of 22 prognostic models of immune cell populations. (A) The results of uni-cox analysis of variance. (B) and (C) Results of lasso regression analysis. (D) Multi-cox analysis result of immune cell population obtained from lasso regression analysis. (E) The nomogram of the prognostic model of immune cell population. (F) ROC curves of patients with OC at 1, 3, and 5 years (TCGA OC dataset and GEO dataset). (G) Kaplan-Meier curves of patients with OC (TCGA OC dataset and GEO dataset).

**Supplementary Table Legends**

**Supplementary Table 1:** Basic information of series used for estimating fractions of tumor microenvironment cells

**Supplementary Table 2:** Relative Fractions of tumor microenvironment cells of ovarian cancer patients with overall survival informations

**Supplementary Table 3:** ICI signature genes A and B

**Supplementary Table 4:** ICI signature genes A and B（PCA algorithm）

**Supplementary Table 5:** GO and KEGG enrichment analyses of ICI signature genes A and B

**Supplementary Table 6:** The results of GSEA

**Supplementary Table 7:** The TMB score(patients from TCGA database )

**Supplementary Table 8:** Uni-factor COX, lasso, and multi-factor COX analysis and risk scores for prognostic models of immune-related genes

**Supplementary Table 9:** Risk score (prognostic models of immune-related genes; GSE140082)

**Supplementary Table 10:** Uni-factor COX, lasso, multi-factor COX analysis and risk scores for prognostic models of immune-related genes, ICI, and TMB
